# Clinical features and prognostic factors of elderly patients with metastatic pancreatic cancer: a population-based study

**DOI:** 10.18632/aging.202570

**Published:** 2021-02-26

**Authors:** Tao Lianyuan, Li Deyu, Yu Haibo, Dong Yadong, Tian Guanjing

**Affiliations:** 1 Department of Hepatobiliary Surgery, Henan Provincial People's Hospital, People's Hospital of Zhengzhou University, School of Clinical Medicine, Henan University, Zhengzhou 450003, Henan, China

**Keywords:** elderly patients, pancreatic cancer, metastasis, prognostic factors, survival

## Abstract

The aim of this study was to evaluate the clinical features and prognostic factors of elderly patients with metastatic pancreatic cancer. Patients diagnosed with metastatic pancreatic cancer between 2004 and 2014 were identified from the Surveillance Epidemiology and End Results database. Clinical characteristics and prognostic factors in elderly patients with metastatic pancreatic cancer were examined. A total of 10784 metastatic pancreatic cancer patients between 65 and 80 years old were included and divided into three age groups. Elderly metastatic pancreatic cancer patients differed from younger patients in many aspects, including marital status, race, sex, T stage, N stage, treatment regimen, prognosis, cause of death, and metastatic characteristics (P<0.001). An analysis of prognostic factors showed that chemotherapy, as the main treatment for elderly patients, can significantly improve prognosis, while surgery can improve the prognosis of patients between 65 and 80 years old. Other factors, including sex, marital status, T stage, and site of metastasis, had different effects on patients in different age groups. Elderly patients with metastatic pancreatic cancer are a special group of individuals whose clinical characteristics and prognostic factors are different from those of younger patients, and these patients require special treatment and attention.

## INTRODUCTION

As the world's population structure is increasingly aging, the incidence of cancer is growing [1]. It is expected that by 2030, seventy percent of malignancies and 85 percent of tumor-related deaths will occur in elderly people (over 65 years) [2]. As a highly malignant tumor, pancreatic cancer (PC) is the fourth leading cause of tumor-related death and is expected to become the second leading cause by 2030 in the US [3, 4]. Due to the lack of clinical manifestations in the early stage, most PC patients are diagnosed at an advanced stage accompanied by metastasis [5, 6]. Although the overall incidence of PC among all age groups is only 11.7 percent, the incidence of PC is 66.4 percent in patients over 65 years old and 91.1 percent in patients over 80 years old [7]. Therefore, treating elderly patients with metastatic pancreatic cancer (mPC), an increasingly expanding group, is going to be a serious clinical challenge.

It has been widely recognized that elderly patients’ internal environment (chronic inflammation and immune system dysfunction) is more likely to induce cancer under the stimulation of carcinogens [8, 9]. Moreover, it has been reported that age is a significant negative prognostic factor for PC [10], and the immune system of elderly patients plays a key role in the development of PC [11]. Therefore, elderly patients may represent a distinct subgroup that needs more targeted clinical management plans [12]. At present, surgery is not recommended for pancreatic cancer patients with distant metastases, especially elderly patients, who are more likely to receive chemotherapy, radiotherapy and other nonsurgical treatments [7, 13]. The aim of this study was to explore the clinical characteristics and prognostic factors of elderly patients with mPC.

## RESULTS

### Patient characteristics

We used X-tile software to divide the patients who diagnosed between 2004 and 2014 by age into three groups. The results indicated that the ages of 65 and 80 years old were appropriate cutoff values for age at diagnosis (Supplementary Figure 1). A total of 10,784 patients were enrolled in this study, including 3681 aged under 65 years, 4415 between 65 and 80 years, and 2688 over 80 years. The clinical characteristics of the mPC patients stratified by age are presented in Table 1. Approximately half of the patients were married (N=5329, 49.4%). Most patients were white (N=8560, 79.4%), and male patients accounted for 51.7% of all patients (N=5573). In total, 551 patients (5.1%) were treated surgically, and 3883 (36%) received chemotherapy. The collection of metastatic location data in the SEER database began in 2010; thus, detailed information about the metastatic sites of 5463 patients was included from 2010 to 2014.

**Table 1:** Comparison of characteristics of metastatic pancreatic cancer patients among different age groups.

**Age group**		**All patients**	**Age<65**	**Age ≥65 and <80**	**Age ≥80**	**P value**
**Features of Patients (2004-2014)**		10784(100.0%)	3681(100%)	4415(100%)	2688(100%)	
**Marital status**	Others	5455(50.6%)	1748(47.5%)	2016(45.7%)	1691(62.9%)	<0.001
	Married	5329(49.4%)	1933(52.5%)	2399(54.3%)	997(37.1%)	
**Race**	Others	2224(20.6%)	902(24.5%)	885(20%)	437(16.3%)	<0.001
	White	8560(79.4%)	2779(75.5%)	3530(80%)	2251(83.7%)	
**Sex**	Male	5573(51.7%)	2191(59.5%)	2288(51.8%)	1094(40.7%)	<0.001
	Female	5211(48.3%)	1490(40.5%)	2127(48.2%)	1594(59.3%)	
**T stage**	T0	515(4.8%)	181(4.9%)	218(4.9%)	116(4.3%)	<0.001
	T1	103(1.0%)	32(0.9%)	47(1.1%)	24(0.9%)	
	T2	766(7.1%)	248(6.7%)	316(7.2%)	202(7.5%)	
	T3	1032(9.6%)	390(10.6%)	422(9.6%)	220(8.2%)	
	T4	1204(11.2%)	472(12.8%)	488(11.1%)	244(9.1%)	
	Tx	7164(66.4%)	2358(64.1%)	2924(66.2%)	1882(70%)	
**N stage**	N0	3625(33.6%)	1186(32.2%)	1552(35.2%)	887(33%)	<0.001
	N1	2125(19.7%)	927(25.2%)	823(18.6%)	375(14%)	
	Nx	5034(46.7%)	1568(42.6%)	2040(46.2%)	1426(53.1%)	
**Surgery**	No	10123(93.9%)	3425(93%)	4137(93.7%)	2561(95.3%)	<0.001
	Yes	551(5.1%)	220(6%)	241(5.5%)	90(3.3%)	
	Unknown	110(1.0%)	36(1%)	37(0.8%)	37(1.4%)	
**Radiation**	No	10350(96.0%)	3488(94.8%)	4232(95.9%	2630(97.8%)	<0.001
	Yes	434(4.0%)	193(5.2%)	183(4.1%)	58(2.2%)	
**Chemotherapy**	No	6901(64.0%)	1896(51.5%)	2722(61.7%)	2283(84.9%)	<0.001
	Yes	3883(36.0%)	1785(48.5%)	1693(38.3%)	405(15.1%)	
**OS**	Live	595(5.5%)	305(8.3%)	212(4.8%)	78(2.9%)	<0.001
	Dead	10189(94.5%)	3376(91.7%)	4203(95.2%)	2610(97.1%)	
**CSS**	Others	2104(19.5%)	632(17.2%)	849(19.2%)	623(23.2%)	<0.001
	Dead of PC	8680(80.5%)	3049(82.8%)	3566(80.8%)	2065(76.8%)	
**Metastases of Patients (2010-2014)**		5463(100.0%)	1769(100%)	2238(100%)	1456(100%)	
**Bone metastasis**	No	4318(79.0%)	1416(80%)	1782(79.6%)	1120(76.9%)	<0.001
	Yes	480(8.8%)	189(10.7%)	202(9%)	89(6.1%)	
	Unknown	665(12.2%)	164(9.3%)	254(11.3%)	247(17%)	
**Brain metastasis**	No	4702(86.1%)	1563(88.4%)	1945(86.9%)	1194(82%)	<0.001
	Yes	67(1.2%)	26(1.5%)	29(1.3%)	12(0.8%)	
	Unknown	694(12.7%)	180(10.2%)	264(11.8%)	250(17.2%)	
**Liver metastasis**	No	1217(22.3%)	386(21.8%)	482(21.5%)	349(24%)	<0.001
	Yes	3892(71.2%)	1293(73.1%)	1631(72.9%)	968(66.5%)	
	Unknown	354(6.5%)	90(5.1%)	125(5.6%)	139(9.5%)	
**Lung metastasis**	No	3627(66.4%)	1220(69%)	1478(66%)	929(63.8%)	<0.001
	Yes	1165(21.3%)	381(21.5%)	495(22.1%)	289(19.8%)	
	Unknown	671(12.3%)	168(9.5%)	265(11.8%)	238(16.3%)	

OS: overall survival; CSS: cancer-specific survival.

On the one hand, compared with the number of mPC patients under 65 years old, more mPC patients between 65 and 80 years old were married, were white, were female, had T1 and T2 stage disease, and had N0 stage disease and were less likely to be treated with surgery, radiation, or chemotherapy (all P <0.001). Although the liver is the organ that most commonly develops metastasis, elderly patients have a greater chance of developing lung metastasis than younger patients and are less likely to develop metastasis in the liver, brain, or bone (all P <0.001). Detailed information is shown in Table 1. On the other hand, there was a large proportion of mPC patients older than 80 years who were unmarried (which contrasts with mPC patients between the ages of 65 and 80 years), were white, were female, had T1 and T2 stage disease, and had N0 stage disease and were less likely to be treated with surgery, radiation and chemotherapy (all P <0.001). Moreover, it may be more difficult to identify metastatic sites in elderly patients, including the liver, lung, brain, or bone (all of these sites have a lower diagnosis rate in elderly patients than in younger patients; all P <0.001, Table 1).

The analysis also indicated that elderly mPC patients had a higher mortality rate at the follow-up deadline but a lower tumor-specific mortality rate than younger patients (all P <0.001). Of the 10784 patients, mortality occurred in 10189 (94.5% of 10784) at the end of follow-up. Among them, 8680 (80.5% of 10784) patients died due to pancreatic cancer. Regarding CSS, the cancer-specific mortality rates were 82.8% in the under 65-year-old group, 80.8% in the 65- to 80-year-old group, and 76.8% in the over 80-year-old group. Regarding OS, the mortality rates were 91.7%, 95.2% and 97.1% in the three age groups, respectively.

### Prognostic factors of mPC patients between 65 and 80 years old

**Figure 1:**
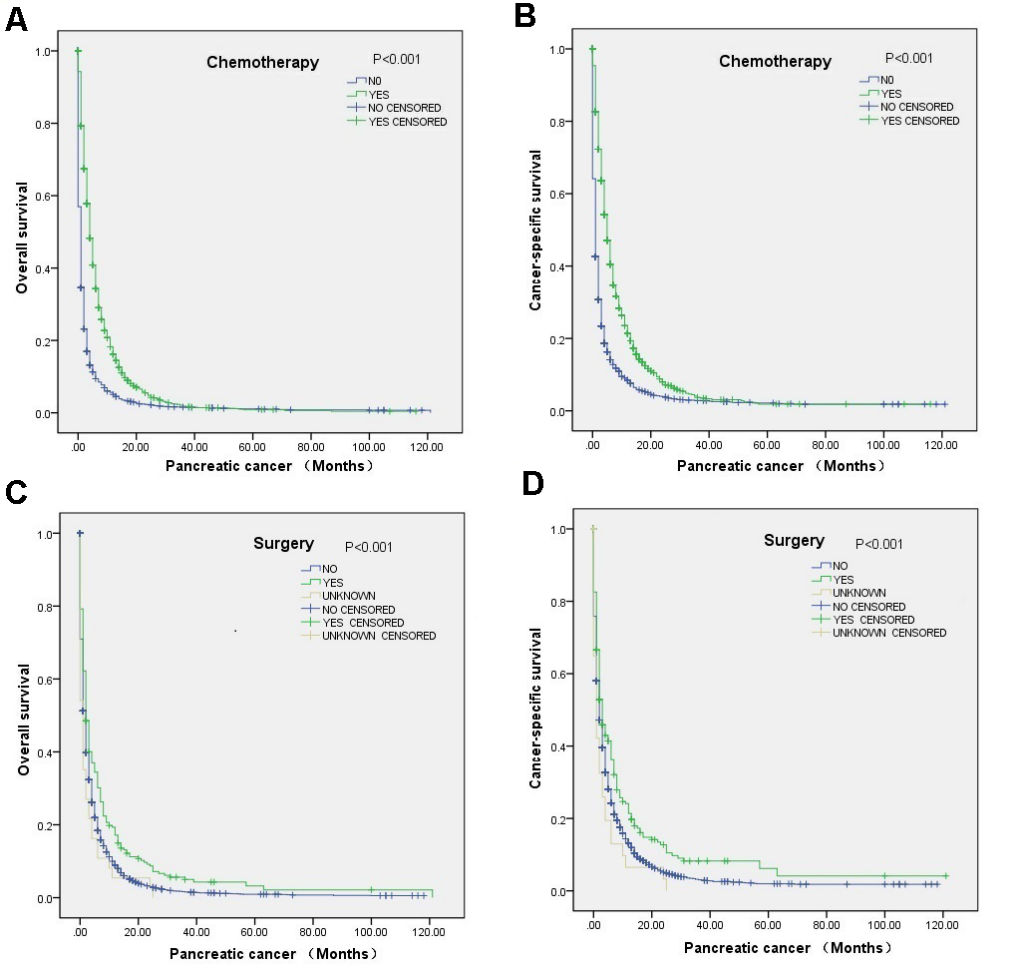
Survival curves of elderly mPC patients between 65 and 80 years old who received chemotherapy or surgery according to the log-rank test: (**A**) OS (P<0.001) and (**B**) CCS (P<0.001) for the chemotherapy, and (**C**) OS (P<0.001) and (**D**) CCS (P<0.001).for the surgery.

Multivariate Cox regression analysis revealed that surgical resection was associated with better OS (HR=0.70, 95% CI=0.57-0.85) and CSS (HR=0.72, 95% CI=0.58-0.90) and that chemotherapy was also associated with better OS (HR=0.45, 95% CI=0.41-0.49) and CSS (HR=0.43, 95% CI=0.39-0.48) (Table 2). The correlations of chemotherapy or surgical resection with OS and CSS according to the log-rank test was also revealed through the survival curve (Figure 1). Moreover, the results demonstrated that factors associated with poor OS included being unmarried, T0 stage disease and lung metastasis. In addition, poor CSS tended to occur in patients with T0 stage disease or lung metastasis. The detailed patient characteristics are shown in Table 2.

**Table 2:** Multivariate analysis of overall survival and cancer-specific survival in metastatic pancreatic cancer with age above 65 and under 80 years old.

**Variables**		**OS**			**CSS**		
		**HR**	**(95%CI)**	**P value**	**HR**	**(95%CI)**	**P value**
**Marital status**	Others		1 (Referent)			1 (Referent)	
	Married	0.90	(0.83-0.99)	0.027	0.95	(0.86-1.06)	0.345
**Race**	Others		1 (Referent)			1 (Referent)	
	White	0.96	(0.86-1.07)	0.467	0.94	(0.83-1.07)	0.327
**Sex**	Male		1 (Referent)			1 (Referent)	
	Female	0.96	(0.88-1.05)	0.338	1.05	(0.95-1.17)	0.348
**T stage**	T0		1 (Referent)			1 (Referent)	
	T1	0.60	(0.40-0.89)	0.012	0.57	(0.35-0.93)	0.025
	T2	0.74	(0.59-0.95)	0.015	0.76	(0.57-1.00)	0.053
	T3	0.85	(0.67-1.08)	0.183	0.85	(0.64-1.12)	0.254
	T4	0.94	(0.75-1.18)	0.573	0.98	(0.75-1.28)	0.880
	Tx	0.81	(0.66-0.99)	0.036	0.81	(0.64-1.03)	0.084
**N stage**	N0		1 (Referent)			1 (Referent)	
	N1	0.96	(0.85-1.08)	0.496	0.96	(0.83-1.11)	0.580
	Nx	0.99	(0.89-1.10)	0.817	1.05	(0.93-1.19)	0.438
**Surgery**	No		1 (Referent)			1 (Referent)	
	Yes	0.70	(0.57-0.85)	<0.001	0.72	(0.58-0.90)	0.005
	Unknown	1.18	(0.76-1.84)	0.466	1.34	(0.83-2.17)	0.236
**Radiation**	No		1 (Referent)			1 (Referent)	
	Yes	0.88	(0.69-1.11)	0.282	0.83	(0.62-1.10)	0.193
**Chemotherapy**	No		1 (Referent)			1 (Referent)	
	Yes	0.45	(0.41-0.49)	<0.001	0.43	(0.39-0.48)	<0.001
**Bone metastasis**	No		1 (Referent)			1 (Referent)	
	Yes	1.05	(0.88-1.24)	0.592	1.02	(0.83-1.25)	0.830
	Unknown	1.12	(0.77-1.62)	0.560	1.03	(0.67-1.58)	0.894
**Brain metastasis**	No		1 (Referent)			1 (Referent)	
	Yes	1.27	(0.85-1.88)	0.241	1.26	(0.79-2.01)	0.331
	Unknown	0.96	(0.66-1.41)	0.848	0.99	(0.64-1.52)	0.947
**Liver metastasis**	No		1 (Referent)			1 (Referent)	
	Yes	1.09	(0.98-1.22)	0.130	1.10	(0.96-1.25)	0.170
	Unknown	1.05	(0.82-1.36)	0.692	1.03	(0.76-1.38)	0.867
**Lung metastasis**	No		1 (Referent)			1 (Referent)	
	Yes	1.18	(1.05-1.32)	0.004	1.21	(1.06-1.38)	0.004
	Unknown	0.93	(0.75-1.15)	0.486	1.02	(0.80-1.30)	0.891

OS: overall survival; CSS: cancer-specific survival; HR: hazard ratio; CI: confidence interval.

### Prognostic factors of mPC patients aged above 80 years old

**Figure 2:**
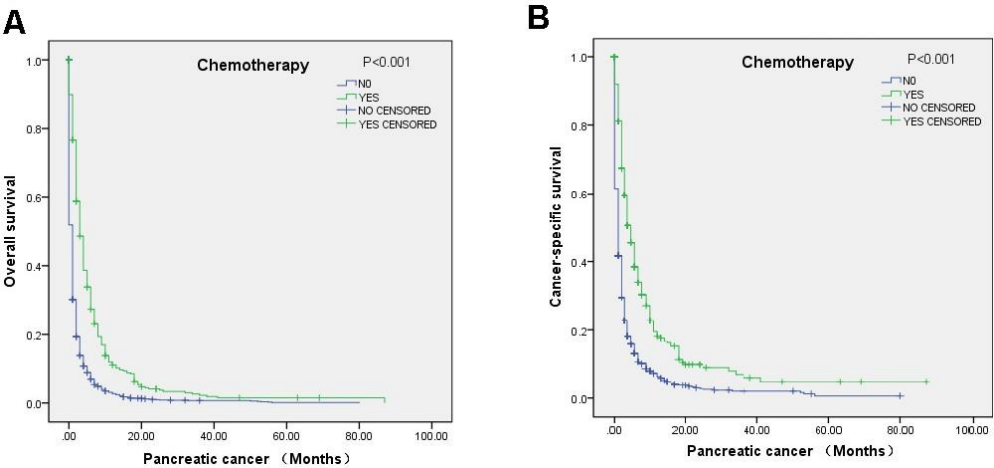
Survival curves of elderly mPC patients over 80 years old who received chemotherapy according to the log-rank test: (**A**) OS (p<0.001) and (**B**) CSS (P<0.001).

Multivariate Cox regression analysis indicated that chemotherapy was associated with improved OS (HR=0.45, 95% CI=0.41-0.49) and CSS (HR=0.43, 95% CI=0.39-0.48), and radiation showed a positive effect on OS (HR=0.64, 95% CI=0.45-0.92) but not CCS (Table 3). The relationships of chemotherapy and surgical resection with OS and CCS according to the log-rank test were also revealed through survival curves (Figure 2). Moreover, the analysis indicated that the factors associated with poor OS included male sex and liver metastasis (Table 3).

**Table 3:** Multivariate analysis of overall survival and cancer-specific survival in metastatic pancreatic cancer with age above 80 years old.

**Variables**		**OS**			**CSS**		
		**HR**	**(95%CI)**	**P value**	**HR**	**(95%CI)**	**P value**
**Marital status**	Others		1 (Referent)			1 (Referent)	
	Married	0.95	(0.84-1.07)	0.364	1.02	(0.88-1.18)	0.830
**Race**	Others		1 (Referent)			1 (Referent)	
	White	1.08	(0.93-1.25)	0.304	1.01	(0.85-1.21)	0.904
**Sex**	Male		1 (Referent)			1 (Referent)	
	Female	0.88	(0.79-0.99)	0.037	1.05	(0.91-1.22)	0.476
**T stage**	T0		1 (Referent)			1 (Referent)	
	T1	0.75	(0.40-1.38)	0.354	0.73	(0.33-1.63)	0.440
	T2	1.08	(0.81-1.45)	0.593	1.18	(0.81-1.72)	0.382
	T3	0.93	(0.69-1.25)	0.638	1.04	(0.71-1.52)	0.847
	T4	1.03	(0.76-1.38)	0.864	1.14	(0.78-1.66)	0.500
	Tx	1.06	(0.82-1.36)	0.674	1.18	(0.85-1.63)	0.333
**N stage**	N0		1 (Referent)			1 (Referent)	
	N1	1.06	(0.9-1.240)	0.47	1.2	(0.99-1.46)	0.063
	Nx	0.94	(0.82-1.07)	0.326	1.01	(0.86-1.19)	0.911
**Surgery**	No		1 (Referent)			1 (Referent)	
	Yes	0.78	(0.58-1.05)	0.101	0.85	(0.60-1.21)	0.375
	Unknown	0.88	(0.59-1.32)	0.545	0.96	(0.61-1.5)	0.842
**Radiation**	No		1 (Referent)			1 (Referent)	
	Yes	0.64	(0.45-0.92)	0.014	0.84	(0.56-1.25)	0.380
**Chemotherapy**	No		1 (Referent)			1 (Referent)	
	Yes	0.48	(0.41-0.56)	<0.001	0.43	(0.35-0.53)	<0.001
**Bone metastasis**	No		1 (Referent)			1 (Referent)	
	Yes	0.98	(0.77-1.24)	0.86	0.76	(0.56-1.05)	0.095
	Unknown	1.13	(0.77-1.66)	0.525	1.07	(0.68-1.70)	0.765
**Brain metastasis**	No		1 (Referent)			1 (Referent)	
	Yes	1.42	(0.79-2.58)	0.243	1.48	(0.72-3.04)	0.289
	Unknown	1.11	(0.76-1.62)	0.581	1.28	(0.82-2.00)	0.284
**Liver metastasis**	No		1 (Referent)			1 (Referent)	
	Yes	1.22	(1.07-1.40)	0.003	1.17	(1.00-1.38)	0.055
	Unknown	0.99	(0.77-1.28)	0.934	1.04	(0.77-1.40)	0.814
**Lung metastasis**	No		1 (Referent)			1 (Referent)	
	Yes	1.00	(0.87-1.16)	0.992	1.00	(0.84-1.19)	0.996
	Unknown	0.84	(0.64-1.10)	0.201	0.79	(0.58-1.10)	0.160

OS: overall survival; CSS: cancer-specific survival; HR: hazard ratio; CI: confidence interval.

### Validation set and performance

Patients who diagnosed at 2015 were set as the validation dataset, and detailed patients’ characteristics in different year of diagnosis was listed (Supplementary Table 1). For elderly group. we examined the performance of the prediction final model, both in the development dataset (2010-2014) and the validation dataset (2015) in terms of discrimination, which measured using values of 3-year time-dependent AUC. In the primary cohort, the model showed the discriminative ability in the validation cohort was agree with the development dataset (AUC: 0.699 vs 0.679, Supplementary Figure 2). Calibration was examined by plotting agreement between predicted and observed risks (Supplementary Figure 3). The imaginary line indicates a perfect calibration model in which the predicted probabilities are identical to the actual survival outcomes with the calibration slope of 1.0755 (0.9546 to 1.2117).

## DISCUSSION

According to the characteristics of previously reported age groups [7], the elderly patients in this study were divided into a 65- to 80-year-old group and an over 80-years-old group. The statistical results showed that elderly patients accounted for the majority of all mPC patients (65.9%), especially those between 65 and 80 years old (40.9%). A comparison of the clinical characteristics among patients of different ages revealed that white patients were more likely to have a longer life span, possibly because white patients tend to have more access to medical services [14, 15].

The results also showed that female sex was a favorable prognostic factor for mPC patients over 80 years old, which may be because females tend to pay more attention to their health and make greater use of health-care services than males [16, 17]. In addition, the proportion of patients between 65 and 80 years old who were married was the highest (2399/4415, 54.3%) among all age groups, while the proportion of married patients between 65 and 80 years old was sharply reduced (997/2688, 37.1%), which may be attributed to the higher death rate of their spouses during this age range. Further prognostic analysis indicated that marriage is a favorable prognostic factor for mPC patients between 65 and 80 years old, which agrees with many previous studies [18–21]. The result indicates that elderly people who lack care from a marriage partner have a poor prognosis. Therefore, elderly patients who do not have spouses should be given more attention.

Further study shows that there was an increase in the proportion of patients with T1 and T2 stage disease and a decrease in the proportion of patients with lymph node-negative disease (N0) among elderly patients. This result can be attributed to the frequent routine check-ups of elderly patients, which can allow for the diagnosis of disease at an early stage. Surprisingly, prognostic factor analysis suggested that patients with T0 stage disease between 65 and 80 years old had a better prognosis than those with Tn stage disease. Metastases that develop at an earlier T stage have a stronger ability to invade and metastasize; therefore, the tumor is more malignant, and the prognosis is correspondingly worse. This result is consistent with a previous study [20].

The older the cancer patient is, the more basic diseases he or she has and the more likely he or she will die from these diseases [7, 13], which was confirmed by our results. The present study showed that the overall mortality rate was higher among older patients; however, the tumor-specific mortality rate decreased gradually with increasing age. Such a result suggests that attention should be paid not only to the treatment of tumors in aging patients, especially in those over 80 years old, but also to the systemic conditions and basic diseases of these patients. Therefore, we tend to use the CSS as the prognostic indictor in the elderly group.

Moreover, the analysis of metastatic sites (collected from 2010 to 2014) suggested that the most common target organs for metastasis are the liver, lung, bone, and brain. A comparison among the age groups suggested that as age increases, except for the increase in lung metastasis between 65 and 80 years old, the metastasis rate in all sites showed a decreasing trend. This may be because tumors in elderly patients are often less aggressive than those in young patients. The liver and lungs are vital organs in the human body. Once metastasis occurs in these sites, patients may have a worse prognosis. Similarly, the present analysis also indicated that lung metastasis, which is related to both OS and CCS, is a poor prognostic factor for patients between 65 and 80 years old, while liver metastasis is related to OS in those over 80 years old.

Data on treatment showed that the older the patient is, the less likely he or she is to receive treatment, including surgery, radiation, and chemotherapy. Nonetheless, chemotherapy was the most common form of treatment, followed by surgery and radiation. Further analysis suggested that chemotherapy has the greatest therapeutic benefit for patients between 65 and 80 years old or older. Chemotherapy has been proven to be the primary treatment for mPC [22–25]. Gemcitabine alone or in combination with other chemotherapeutic drugs and the FOLFIRINOX regimen (leucovorin, fluorouracil, irinotecan, and oxaliplatin) are recommended according to the patient’s performance status as well as comorbidity profile [26]. Surgical treatment is not recommended for mPC, especially in elderly patients, according to clinical guidelines. However, surgery has been demonstrated to improve the prognosis of mPC patients [20]. The present analysis also indicated that patients between 65 and 80 years old may still benefit from surgery, but not those over 80 years old (which may be due to their physical condition and inability to tolerate surgery). This result is in agreement with those for other metastatic cancers, such as those originating from renal cells [27], the colorectum [28], and the prostate [29, 30], which have been proven to benefit from surgical treatment of the primary tumor. Although radiotherapy has a positive effect on OS among those between 65 and 80 years old, this treatment failed to show a correlation with CCS and had no association with death in patients over 80 years old. Such a result should be attributed to the lower sensitivity of pancreatic cancer to radiotherapy in elderly patients [23, 31]. During the validation process, its C-index value of prediction model was almost similar to the development dataset (AUC: 0.699 vs 0.679, Supplementary Figure 2). Moreover, the calibration ability detection indicates a perfect condition that the predicted probabilities are identical to the actual outcome with a calibration slope of 1.0755 (0.9546 to 1.2117).

The present study utilized a population-based cohort, which has the advantage of minimizing selection bias in comparisons. However, there are still some limitations, such as the relatively incomplete clinical information of many patients. For example, data about the sites of metastasis were collected only from 2010 to 2014, and patients enrolled before this period lacked relevant data. A large proportion of data regarding T stage, N stage, or metastatic sites were recorded as not otherwise specified or unknown. Information on the specific chemotherapy regimen used was not available. Because of these limitations, further evaluations of treatment in elderly patients were hindered, and we cannot rule out an alternative explanation for some of our findings. The remaining questions could be answered in future studies with more detailed information regarding clinical characteristics and treatment protocols. Our findings may be useful to establish a treatment policy for elderly mPC patients.

## CONCLUSIONS

Elderly mPC patients differed from younger patients in many aspects. Chemotherapy, as the main treatment for elderly patients, can significantly improve their prognosis. Therefore, elderly patients with mPC are a special group of patients whose clinical characteristics and prognostic factors are different from those of young patients, and these patients require special treatment and attention. Chemotherapy is the most reasonable treatment and can improve the prognosis of elderly patients.

## MATERIALS AND METHODS

### Patient cohort

The data examined in the present study were retrieved from the SEER-18 registry of the National Cancer Institute through SEER*Stat Software Version 8.3.5 software to query data from 18 SEER registries. As a publicly available database, the SEER database contains deidentified data; therefore, this study did not need approval from the institutional review board. Patients with a primary site of ‘pancreas’ between January 1, 2004, and December 31, 2014 were identified. The inclusion criteria were as follows: patients with International Classification of Diseases for Oncology, third edition (ICD-O-3) codes 8010, 8020, 8140, 8141 and 8144, and American Joint Committee on Cancer (AJCC) stage (6^th^ edition) IV. Patients with unknown sites of cancer metastasis or unknown age and those who lacked survival data were excluded. The grouping of all patients by age was checked through X-tile software v3.6.1 (Yale University, New Haven, CT, USA), which was utilized to determine the optimal cutoff values [32]. Patients who diagnosed at 2015 were also collect at similar condition for validation.

### Data collection

Information collected from each patient included age, race, sex, marital status, primary tumor site, T stage, N stage, M stage, surgical resection of the primary site, chemotherapy recode, cause-specific death classification, survival time, and vital status. Cancer-specific survival (CSS) and OS (overall survival) were defined as the time between diagnosis and death from mPC and between diagnosis and death from any cause, respectively. Detailed information on systematic treatment is not provided in the SEER database.

### Statistical analysis

Clinical and demographic features were compared between different groups with the chi-square test. The Kaplan-Meier method with the log-rank test was used to examine CSS and OS. A Cox proportional hazards model was applied for multivariable survival analyses of CSS and OS. P<0.05 was considered statistically significant. IBM SPSS Statistics 22.0 (IBM, Armonk, NY, USA) was applied for all statistical analyses.

A prediction model for the elderly group was developed according to the results of multivariate Cox analysis and using the Empowerstats software (http://www.empowerstats.com/en/). The predictive accuracy of the model was assessed by ROC curve analysis. Besides, 3-year calibrations of the model were performed by comparing the predicted CSS to the observed CSS.

### Ethical approval

This article does not contain any studies with human participants or animals performed by any of the authors.

## Supplementary Material

Supplementary Figures

Supplementary Table 1
