# Supplementary material for: Clinical features and prognostic factors of elderly patients with metastatic pancreatic cancer: a population-based study
Source: Aging (Albany NY). 2021 Feb 26;13(5):7133–46. doi: 10.18632/aging.202570 (PMC7993726; doi:10.18632/aging.202570)
Supplement: Supplementary Figures [file aging-13-202570-s001.pdf]

SUPPLEMENTARY FIGURES

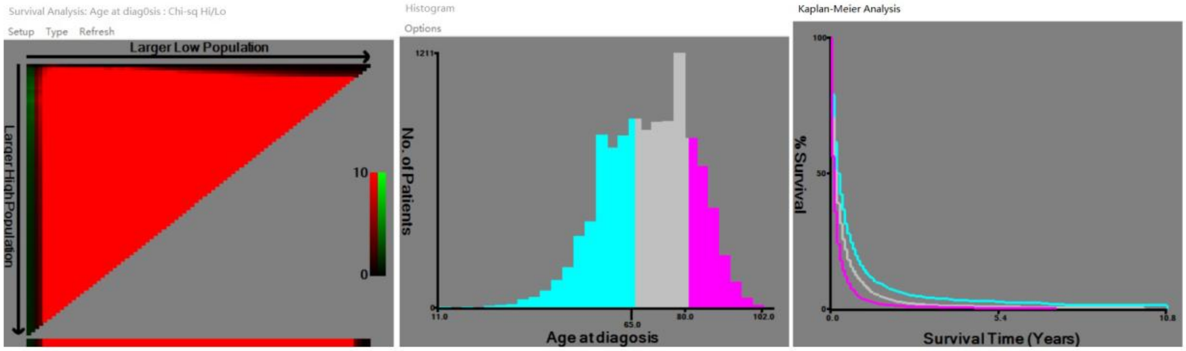

Supplementary Figure 1. Estimation of the cutoff values for age stratification as determined by X-tile software. The ages of 65 and 80 years old were appropriate cutoff values for age at diagnosis.

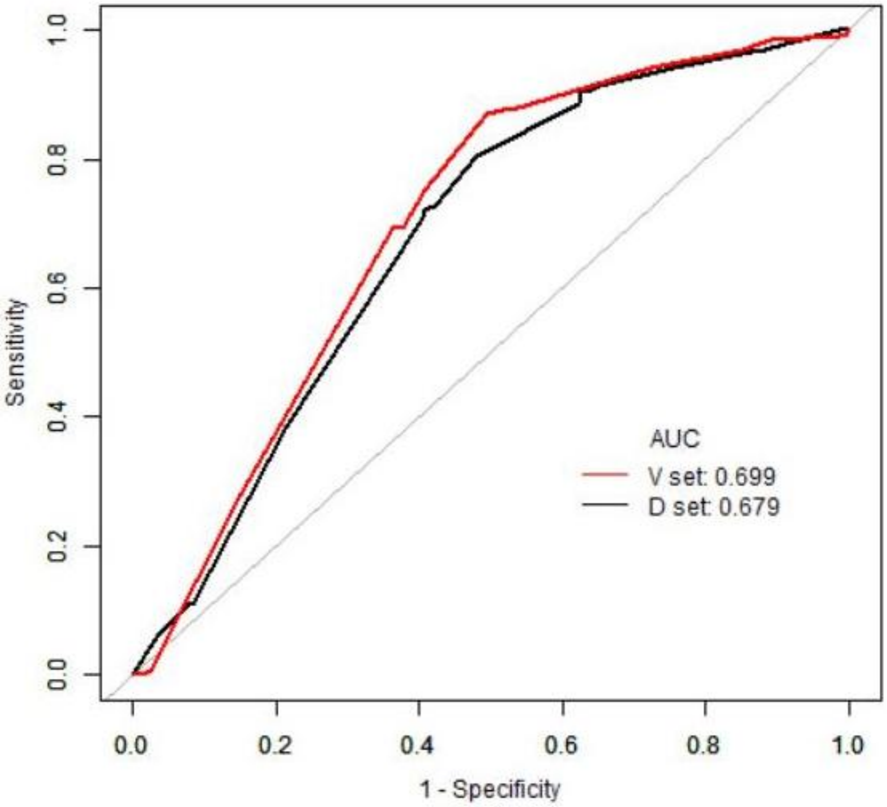

Supplementary Figure 2. Receiver operating characteristic (ROC) curves for development dataset (2010-2014) and validation dataset (2015).

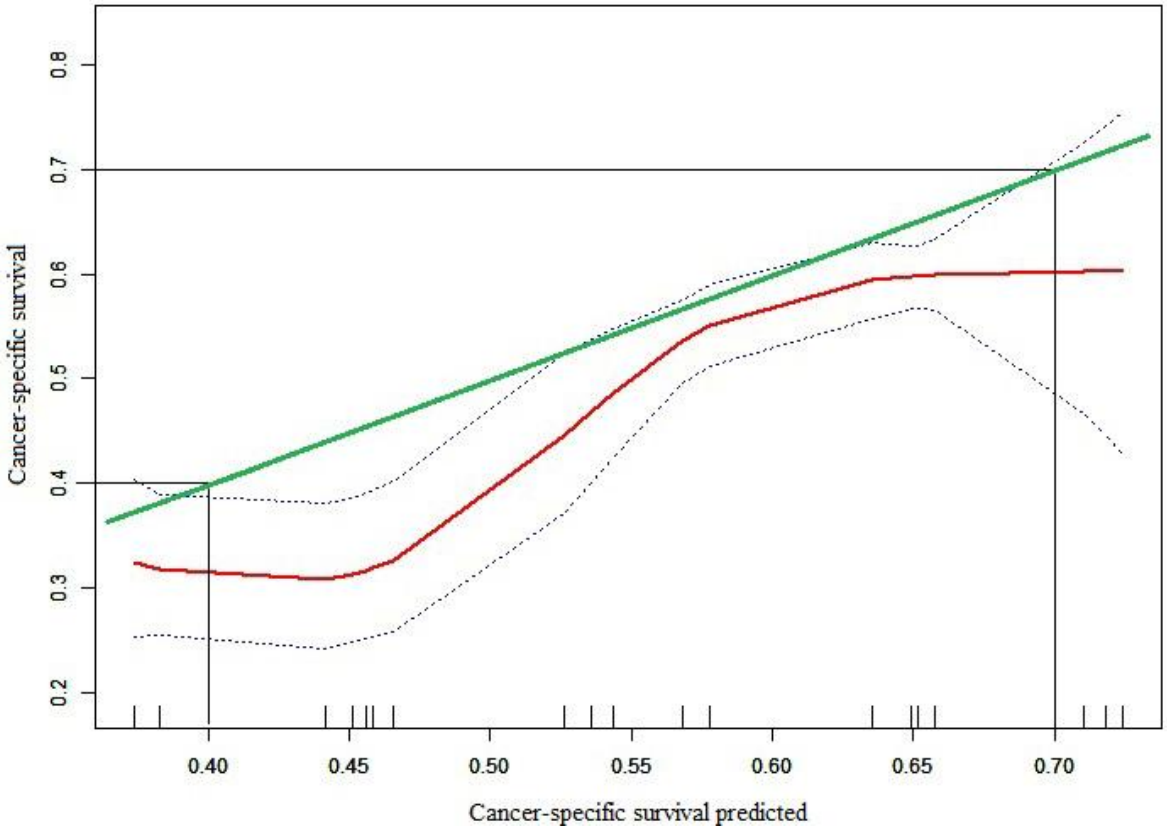

Supplementary Figure 3. Calibration cure for CCS rate plotted against predicted probability of CCS at 3-year after diagnosis.
