# Supplementary material for: Clinical features and prognostic factors of elderly patients with metastatic pancreatic cancer: a population-based study
Source: Aging (Albany NY). 2021 Feb 26;13(5):7133–46. doi: 10.18632/aging.202570 (PMC7993726; doi:10.18632/aging.202570)
Supplement: Supplementary Table 1 [file aging-13-202570-s002.pdf]

SUPPLEMENTARY TABLE

Supplementary Table 1. Characteristic of patients with metastatic pancreatic cancer.

Year of diagnosis	2004-2009	2010-2014	2015
**Features of Patients**	5321(100.0%)	5463(100.0%)	5760(100.0%)
**Marital status**			
**Other**	2625 (49.33%)	2830 (51.80%)	2681 (46.55%)
**Married**	2606 (50.67%)	2633 (48.20%)	3079 (53.45%)
**Age**			
**<65**	1912 (35.93%)	1769 (32.38%)	2018 (35.03%)
**≥65 and <80**	2177 (40.91%)	2238 (40.97%)	2606 (45.24%)
**≥80**	1232 (23.15%)	1456 (26.65%)	1136 (19.72%)
**Race**			
**Other**	1106 (20.79%)	1118 (20.46%)	1227 (21.30%)
**White**	4215 (79.21%)	4345 (79.54%)	4533 (78.70%)
**Sex**			
**Male**	2712 (50.97%)	2861 (52.37%)	3055 (53.04%)
**Female**	2609 (49.03%)	2602 (47.63%)	2705 (46.96%)
**T stage**			
**T0**	232 (4.36%)	283 (5.18%)	61 (1.06%)
**T1**	40 (0.75%)	63 (1.15%)	155 (2.69%)
**T2**	289 (5.43%)	477 (8.73%)	1535 (26.65%)
**T3**	479 (9.00%)	553 (10.12%)	1574 (27.33%)
**T4**	590 (11.09%)	614 (11.24%)	886 (15.38%)
**Tx**	3691 (69.37%)	3473 (63.57%)	1549 (26.89%)
**N stage**			
**N0**	1577 (29.64%)	2048 (37.49%)	2796 (48.54%)
**N1**	883 (16.59%)	1242 (22.73%)	1972 (34.24%)
**Nx**	2861 (53.77%)	2173 (39.78%)	992 (17.22%)
**Surgery**			
**No**	4994 (93.85%)	5129 (93.89%)	5731 (99.50%)
**Yes**	285 (5.36%)	266 (4.87%)	10 (0.17%)
**Unknown**	42 (0.79%)	68 (1.24%)	19 (0.33%)
**Radiation**			
**No**	5101 (95.87%)	5249 (96.08%)	5542 (96.22%)
**Yes**	220 (4.13%)	214 (3.92%)	218 (3.78%)
**Chemotherapy**			
**No**	3407 (64.03%)	3494 (63.96%)	2962 (51.42%)
**Yes**	1914 (35.97%)	1969 (36.04%)	2798 (48.58%)
**OS**			
**Live**	89 (1.67%)	506 (9.26%)	2283 (39.64%)
**Dead**	5232 (98.33%)	4957 (90.74%)	3477 (60.36%)
**CCS**			
**others**	370 (6.95%)	1734 (31.74%)	3091 (53.66%)
**Dead of PC**	4951 (93.05%)	3729 (68.26%)	2669 (46.34%)
**Bone**			
**No**	0 (%)	4318 (79.04%)	5096 (88.47%)
**Yes**	0 (%)	480 (8.79%)	443 (7.69%)
**unknown**	0 (%)	665 (12.17%)	221 (3.84%)
**Brain**			
**No**	0 (%)	4702 (86.07%)	5519 (95.82%)
**Yes**	0 (%)	67 (1.23%)	26 (0.45%)
**unknown**	0 (%)	694 (12.70%)	215 (3.73%)
**Liver**			
**No**	0 (%)	1217 (22.28%)	1160 (20.14%)
**Yes**	0 (%)	3892 (71.24%)	4511 (78.32%)

**unknown**	0 (%)	354 (6.48%)	89 (1.55%)
**Lung**			
**No**	0 (%)	3627 (66.39%)	4252 (73.82%)
**Yes**	0 (%)	1165 (21.33%)	1245 (21.61%)
**unknown**	0 (%)	671 (12.28%)	263 (4.57%)

OS: overall survival; CSS: cancer-specific survival; PC: pancreatic cancer.
